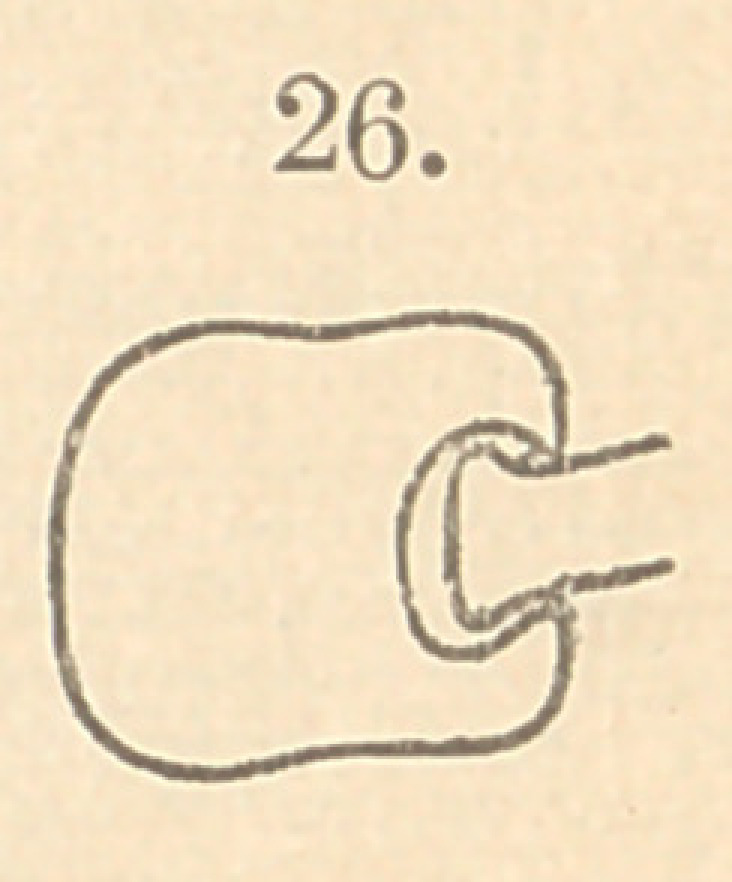# Crown- and Bridge-Work

**Published:** 1892-02

**Authors:** Frederic A. Peeso

**Affiliations:** Philadelphia


### CROWN- AND BRIDGE-WORK.[1]

BY FREDERIC A. PEESO, D.D.S.,[2] PHILADELPHIA.

THERE is probably no branch in the science of dentistry about which there exists a greater diversity of opinion than crown- and bridge-work, and there is certainly no one that has suffered more at the hands of unskilful and incompetent operators. Much has been written and said on this subject, both upholding and condemning it, and strong arguments have been adduced on both sides to prove either its great value or utter worthlessness.

A great many good dentists have been, and still are, strongly opposed to it, but this hostility is decreasing as the work and the methods pursued are becoming more thoroughly understood.

Crown- and bridge-work of the present time is very different from that of a few years ago, and as the people are becoming more enlightened on the subject, the demand for it is increasing, and the work becoming more thorough, and teeth and roots which but a short time since would have been condemned and extracted are now utilized as abutments for anchoring bridges and single crowns, and are giving a great deal more satisfaction than would have been the case had they been removed and plates substituted.

While some condemn the work altogether, others think that a single crown may be all right, or in some cases a very small bridge, but object to extensive pieces, claiming that they cannot stand, but will give way in a short time. If a single crown or a small piece of bridge-work will stand in the mouth, why will not a succession of crowns or an extensive piece stand as well, if the work be equally well done? If the work is used and not abused, it is one of the best things which can be done for our patients.

There is certainly nothing that can be done for them which will please them more, or give more lasting satisfaction, than a denture of this kind.

To most people the thought of wearing a plate is repugnant, but there will not be the same prejudice existing against crown- and bridge-work. It seems nearer to the natural teeth, and where the anchorage is sufficient it is more serviceable, and mastication is more thoroughly performed than it can be by the use of a plate.

---

[1] Read before the Odontological Society of Pennsylvania, December 12, 1891.

[2] Demonstrator of Crown- and Bridge-Work, University of Pennsylvania.

There are unquestionably many failures in the work, and so are there in plates, but as in the case of the latter, a large majority of these failures can be traced to causes which it is possible to remedy, or which if avoided at the beginning, would have made the piece successful.

There are many causes for failures in crown- and bridge-work, the most important of which are insufficient anchorage, improperly-trimmed teeth and roots, poorly-fitted caps and bands, short pins, poor articulation, perforated roots, etc.

The first of these causes to be considered is the anchorage. If this is not sufficient, of course the whole piece is a failure, let the work be never so well done. It is impossible to lay down any rules concerning this, but I will mention a few cases where a serviceable bridge might be placed.

In putting in a full denture it is necessary to have at least four good teeth or roots to serve as abutments. By some this is not thought to be sufficient, but if there are two canines, strong and firm, and two good, strong, solidly set molars, they will carry a bridge for a great many years, and I should not hesitate to place one in such a mouth.

In a case of this kind, the canines should be cut off and Richmond crowns used, and not half caps, as half caps are not suitable for permanent work. If the roots were diseased and could not be brought to a healthy condition, of course there should be no thought of using them. Where the incisors are lost and the canines are standing, often a lasting bridge may be placed, always supposing the teeth strong and healthy.

If the canines stand well apart, and the incisors can be placed nearly straight across the mouth, it will make a strong piece, but if the arch is narrow, and the incisors have to be placed on very much of a curve, it is running a great risk to trust to the canines alone to carry it, as the leverage would probably be so great as to loosen the teeth. Where the six anterior teeth are lost, it is well to give up all thought of a bridge and trust to a plate. In most cases a durable piece can be made running from the canine to the second molar, and sometimes even to the wisdom tooth. The two central incisor roots will carry the two laterals, and the lateral roots the centrals. A loose root does not necessarily preclude the idea of placing a crown. In a great many cases it will yield to treatment and become firm. I have not time to speak further on this subject, but will proceed to the preparation of the mouth.

After being certain that the anchorage is all right, the most important part remaining is the trimming of the teeth and roots. If this is not done properly the whole piece is unsatisfactory and may be considered a failure. I do not think I am in error in saying that in three cases out of four this part of the work is not done at all as it should be.

It will be found that just enough of the tooth has been cut away to allow the passing of the band between it and the adjoining teeth, or if the tooth has broken down so that the band can be passed over, it is not trimmed at all. The cap is put on, and when it is pressed to position the edges stand away from the root or neck of the tooth, cutting into the gum, and being a constant source of irritation, often causing inflammation and loosening of the tooth.

In trimming the teeth, the dentist should always have in mind what the shape of the tooth would be if cut across just below the margin of the gum. The swell should be entirely taken off to nearly one-sixteenth of an inch below the gum line, leaving the sides of the tooth parallel, or slightly larger at the neck, so that when the band is passed over it will hug the tooth closely and not impinge on the gum.

If you will take the trouble to trim a tooth out of the mouth, you will see that in shape it is very different from the majority of those you will see trimmed in the mouth.

Take, for instance, a lower first molar. Looking at it from the buccal side, the swell is very great; starting from just below the gum, it swells out, until where it touches the adjoining teeth it is often from one-quarter to one-third wider than at the neck. Looking from the anterior surface, the swell is not nearly so great, while the masticating surface is oblong. After this tooth has been properly shaped it will be nearly square, with the corners rounded, being slightly narrower at the distal side than at the mesial, owing to the distal root being smaller, and there will be about one-sixteenth of an inch between it and the adjoining teeth. (Fig. 2.) In many instances, after the mesial and distal surfaces are trimmed, it will be necessary to cut only the lingual, as the tooth often leans in towards the tongue, and after this side is cut away the band will pass over the enamel at the buccal side and touch the tooth below the gum. (Fig. 3.) It is often very difficult to trim these teeth on all sides with such wheels as you can get. Probably the most difficult place in the mouth to reach is the anterior surface of the lower molars. If you will take a thin mounted corundum disk and hold it over the spirit-lamp while the engine is running, and as the heat softens it a little press the edges outward, you will form a cup-shaped disk which will do the work nicely. (Fig. 1.) For the lingual and buccal surfaces a small cup-shaped corundum will answer,—No. 11, S. S. White's corundums.

Fig. 1.

After an upper molar has been prepared, it will be found to be altogether different from the lower, being triangular in shape, with the base of the triangle at the buccal side and the apex at the palatal. (Fig. 4.) Of course this may vary at times, and the palatal root be as broad as the two buccal roots, but this is not often so. You will almost invariably find that this tooth has been trimmed about the same as a lower molar, without any regard to the shape of the roots, and that the band, while possibly fitting the tooth at the buccal side, is away from it at the palatal and impinges on the gum. In any of the posterior teeth it will be found that a great deal must be cut from the mesial and distal surfaces, but very little from the buccal and palatal or lingual. The cusps, too, should be ground away, especially where the tooth is to carry one end of a bridge, so as to allow a thick strong metal cusp to be placed on the band, and so form a strong attachment for the rest of the piece.

In making caps for any of these teeth, the band should hug the neck of the tooth tightly, and the contour should be restored till it

Figs. 2. 3. 4. 5. 6.

touches the adjoining teeth; so as to leave no space where the food may find lodgement and annoy the patient.

In preparing the teeth for Richmond crowns, the work is easier and more often properly formed, but here in a great many instances it will be found that the roots are not trimmed at all, or that the work is not more than half done. After the tooth has been cut down to its proper level, if the enamel is taken off it will leave the root of the proper shape for the band to fit nicely, and it is very seldom that it will need any further trimming. (Figs. 5, 6, 7.) This will apply to any of the anterior teeth, or to any tooth where a Richmond crown is to be placed.

The preparation of the canal, too, has a great deal to do with the durability of the work. I think it is the general practice to enlarge the canal without changing its direction at all. In most teeth, if this is done, it does not leave room in front of the pin to place the porcelain without grinding it away or grinding the pin, and thus weakening it. (Fig. 8.) At times the pin is ground entirely off, and the only attachment it has is to the thin floor of the cap. If after having enlarged the canal you will lean the reamer towards the palatal or lingual side of the root, thus sloping the canal in that direction, by bending the pin slightly it will leave plenty of room in front of it to place the facing without weakening it in the least by cutting. (Fig. 9.)

FIGS. 7. 8. 9. 10. 11. 12.

Another prolific cause of weakness is short and small pins. No doubt all of you have been called upon to reset crowns which have become loose for no other reason than that the pins were too short or too small. I have frequently seen a cuspid or a central incisor crown having a pin not more than three-sixteenths or one-quarter of an inch long, and of No. 17 or No. 18 wire, where the root would easily have taken a pin one-half, or even five-eighths, of an inch long and No. 14 or No. 15 wire, and the strain on the tooth would require it. In opening the canal it is always well to ascertain the length of the root first by passing a very fine broach through the apical foramen. You then know just how far you can go, and can make the pin long enough and large enough to give all the strength required. There are very few teeth that will not take a pin at least three-eighths of an inch in length.

Half caps and shoes form very questionable anchorages. They certainly are not suitable for permanent work, as the cement is sure to wash out in time, the piece becomes loose and will have to be reset, and, where the other end is firmly fixed, it is painful and annoying to the patient to have it removed.

I have a bridge in my own mouth that was placed in position two years ago, being anchored at one end with a half cap to the first bicuspid. It is still firm, but if it stays so another year I shall think it will have done all that could have been expected of it.

The strength and lasting qualities of the half cap depends perhaps more than any other on the proper shaping of the tooth. The usual method of trimming the cuspid is to straighten the sides of the tooth and grind away the palatal surface, and here the work stops. The cap is then made and cemented in position. (Fig. 10.) A bridge anchored in this way cannot last, as the force exerted in mastication is sure to loosen it by stretching the band of the half cap in front and forcing it inward over the basilar ridge. My practice is, and I think it is the only way to prepare these teeth, first to trim the sides so that they are nearly parallel. (Fig. 11.) The tooth may be a little wider at the cutting edge than at the neck, as the cap can be sprung over it. Then grind away the palatal surface until it is quite concave. The next cutting is the one on which the stability of the piece entirely depends, and that is the cutting of a groove longitudinally across the back of the tooth, just above the basilar ridge. (Fig. 12.) For this purpose a bur or small corundum wheel can be used.

For the benefit of any who may not have used the half caps I will explain how they are made. The impression should be taken after the tooth has been prepared. After running the model and having it thoroughly dried, cut the plaster away from around the neck of the tooth to nearly one-sixteenth of an inch below the gum line. (Fig. 13.) Then give it several coats of thin sandarach varnish, and after this has dried the model will be hard, and you can proceed to make the cap. First make the band the full height of the tooth, and contour its edges so that it will touch the plaster all around. (Fig. 14.) Cut it out front and back so as to leave these surfaces of the tooth exposed, letting the edges of the band at the inner side extend a little beyond where the tooth has been cut away. (Fig. 15.) Next take a very thin piece of pure gold or platinum of the right width, let it pass over the basilar ridge inside the band. Burnish it carefully into the groove and up the back of the tooth, letting it extend over the cutting edge. (Fig. 16.) Then cover it well with hard wax, remove, invest in sand and plaster, and restore the contour with 18- or 20-carat solder. (Fig. 17.) With a half cap made in this way there is very little strain on the

FIGS. 13. 14. 15. 16. 17. 18.

band in front, and it makes no difference how much pressure is brought to bear, it cannot be forced back over the basilar ridge, and the pressure is directly downward or upward, and nearly over the centre of the tooth.

In the preparation of the bicuspid the same principle will apply. The sides should be made nearly parallel, and the swell should be taken off the inner side of the tooth, so that the band will pass over and touch the tooth all around below the gum. The inner cusp should then be ground off, cutting straight downward at the centre, and sloping it upward towards the point of the cusp. (Fig. 18.) In making the band it should be cut out at the buccal side and even with the top of the inner cusp, as it has been ground. (Fig. 19.) The cusp is made by fitting a small piece of platinum to the band after flowing over it coin, or the same metal as the band, to the required thickness, and soldering it to its place (Figs. 19 and 20), and, as you will see, any pressure has a tendency to crowd the piece more firmly into position. (Fig. 20.)

The trimming of a tooth for a shoe should be the same as for a cuspid half cap, with the difference that the groove need not be so marked, and the cutting edge should be ground off sloping downward or upward towards the gum. (Fig. 21.) The ears of the cap should extend beyond the cutting edge as far as is necessary to restore the length of the tooth. (Fig. 22.) The thin metal over the back should be burnished carefully over the cutting edge, between the ears of the cap (Fig. 22), and this space filled with the same carat gold as the band. (Fig. 23.) It is well with any of the above to double the band in front, thus overcoming the danger of stretching, and making the piece more secure. In trimming these teeth the labial face should always be left intact.

The use of the spud, spur, or bar, as it is variously styled, is advisable in very few cases, and it is a question whether it is safe to rely on it at any time. It should never be used unless it can be

FIGS. 19. 20. 21. 22. 23. 24. 25. 26.

made large, and strongly dovetailed into the tooth (Fig. 24), and then only on very small bridges.

A great many crowns are used without bands, but they are certainly not so safe as a well-made Richmond crown, and should never be used in bridge-work. The cement is likely to wash from under them in time, and they are far more apt to loosen than where the root is properly banded. Then, too, the danger of the root becoming fractured is another argument against their use. Fitting the band is often a somewhat painful operation, but it is quickly done, and the patient will soon forget it if the piece is satisfactory.

After the bridge or crown is completed and fitted in the mouth, the articulation must be corrected. A great many bridges, and a great many plates as well, are placed in the mouth which are really of no practical use, owing to the malarticulation. It will be found in most cases where teeth have been out for some time that those in the opposite jaw have elongated, and have grown so far out that they may touch the gums where the teeth are lost. In such mouths it is impossible to do anything for the patient without first cutting these teeth to their proper level. This is usually a very painful operation for the patient to undergo, and some may object to having it done, but to secure good results and do your patients the greatest possible service it is absolutely necessary to grind them away until you can secure a normal articulation, and the patient will have to bear it. I have never yet had a patient who would not allow me to do what cutting I thought necessary.

I remember one case where a bicuspid had grown out so far as to lock the jaws, and the only movement possible was simply the opening and closing. It was a question either of extracting or grinding away. I chose the latter, devitalizing and cutting the tooth to its proper level.

I removed some pieces of bridge-work this last summer that had been on but a few months, and which were made to accommodate such teeth as these. Of course they were of no use to the patient, but were a constant source of pain and annoyance, as the teeth were not trimmed, nor were the bands properly fitted, and the least forward movement of the lower jaw would throw most of the teeth at least one-eighth of an inch apart.

This work cost the patient twenty-five dollars a tooth. A large part of the bridge-work that is done is as bad, if not worse, than this, and is it any wonder that a great many cry out that bridge-work is a failure?

I removed these pieces, trimmed the teeth as I thought they should be trimmed (although the patient winced a good deal), and put in others, and certainly nothing could be more satisfactory than that work is at the present time. I do not believe in grinding away more than is absolutely necessary, but it is a great mistake, whether putting in a plate or a piece of bridge-work, to let your dislike for cutting the natural teeth or pity for the patient deter you from doing what is really for their benefit.

It may be well to mention one other cause of trouble, and that is the breaking of facings. A great many times this is due to short backings, letting the porcelain extend away beyond the gold, and leaving the whole strain on the pins. (Fig. 25.) If you will let the backings extend to, or just beyond, the cutting edge of the tooth, and then flow solder well up to that point, it will make a stronger piece, as the force of the bite will come partly on the metal. (Fig. 26.)

The last, and a very important operation, is the cementing of the piece. As you are all, probably, more or less familiar with this part of the work I will say very little about it. The piece and teeth, or roots, should be as thoroughly dried as possible. Cement should be put well up into the canals and in all of the caps, and the bridge or crown forced quickly into position. It is well to use plenty of cement, and as the bridge is pushed to its place, the excess will be forced out around the edges of the cap. It is the custom with some to drill holes in the cap to allow the surplus to escape, and then fill these holes with gold, but this is a great mistake, as the caps should be perfectly tight, so as to force the cement to every part.

Now, a few words about working on the model. For this purpose the hardest plaster obtainable should be used. I have given directions about preparing them while speaking on half caps. For all full gold or half caps, I think that the work can be done better and much easier on the model than in the mouth, and certainly it is far more agreeable to the patient. Where you intend to put on a Richmond crown, with a part of the tooth cut below the gum, it is, perhaps, easier to fit the band on the root.

There may be some who doubt the possibility of doing as accurate work on plaster as on the tooth, but you cannot tell what can be done until you have tried it yourself or have seen others do it. I did not think it could be accomplished until convinced of it by Professor Essig, of the University of Pennsylvania, from whom I learned what I know of model work. It certainly requires more skill and care, but after having mastered it, you will work in the mouth only when it is unavoidable.

Where a cap for a Richmond crown is made on the root, if, after taking the impression, a little soft wax is flowed around the inside of the band and on the pin, it will not be necessary to run the model of sand and plaster, but plaster alone may be used. After the cast has become hard, the cap may be taken off with a pair of heated pliers, and the wax burned away. It may then be replaced, and when the crown is ready to invest for soldering, it can easily be removed without having to be cut out, and there will still remain a perfect model on which, if it should be required, another crown could be made.

A volume remains to be written on this subject, and this paper but touches lightly on a few of the most important points. It is essentially a work of details, and it is by giving careful attention to these that the whole is made a success. Where the conditions are favorable and the work is thoroughly done, it is one of the very best things that you can do for your patients. Where the reverse is the case, it is one of the very worst, and the mouth might better have been left untouched.

## Figures and Tables

**Fig. 1. f1:**
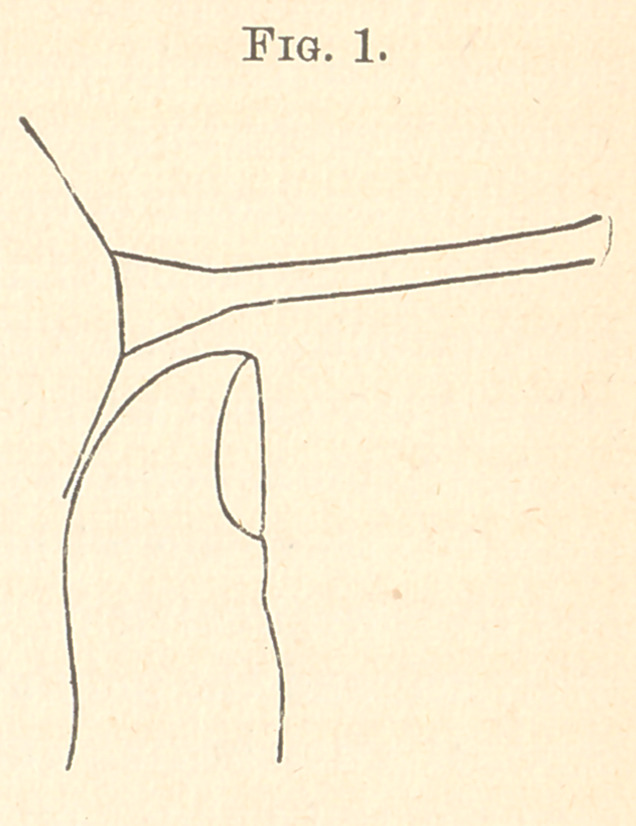


**Fig. 2. f2:**
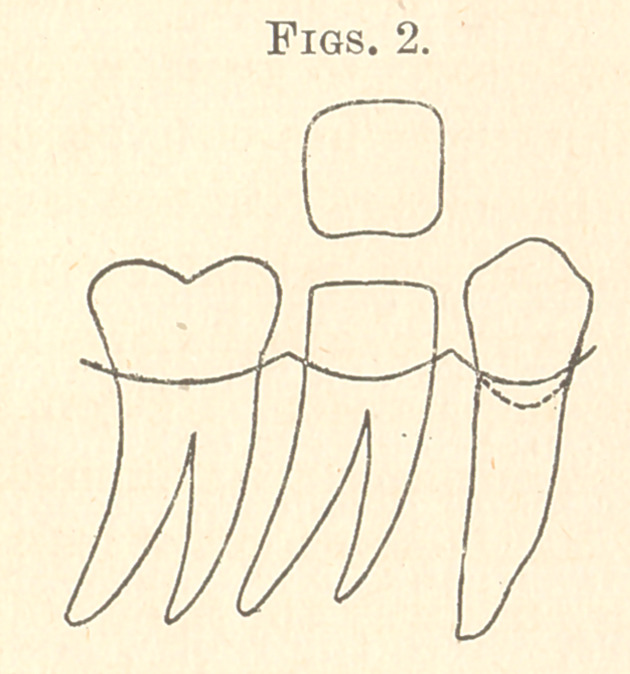


**Fig. 3. f3:**
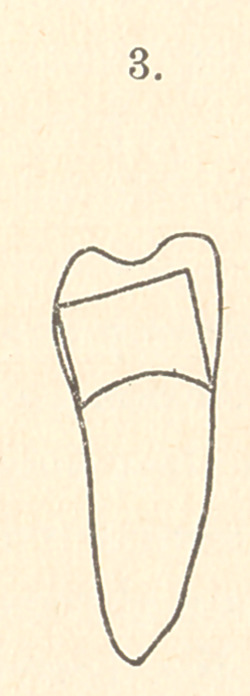


**Fig. 4. f4:**
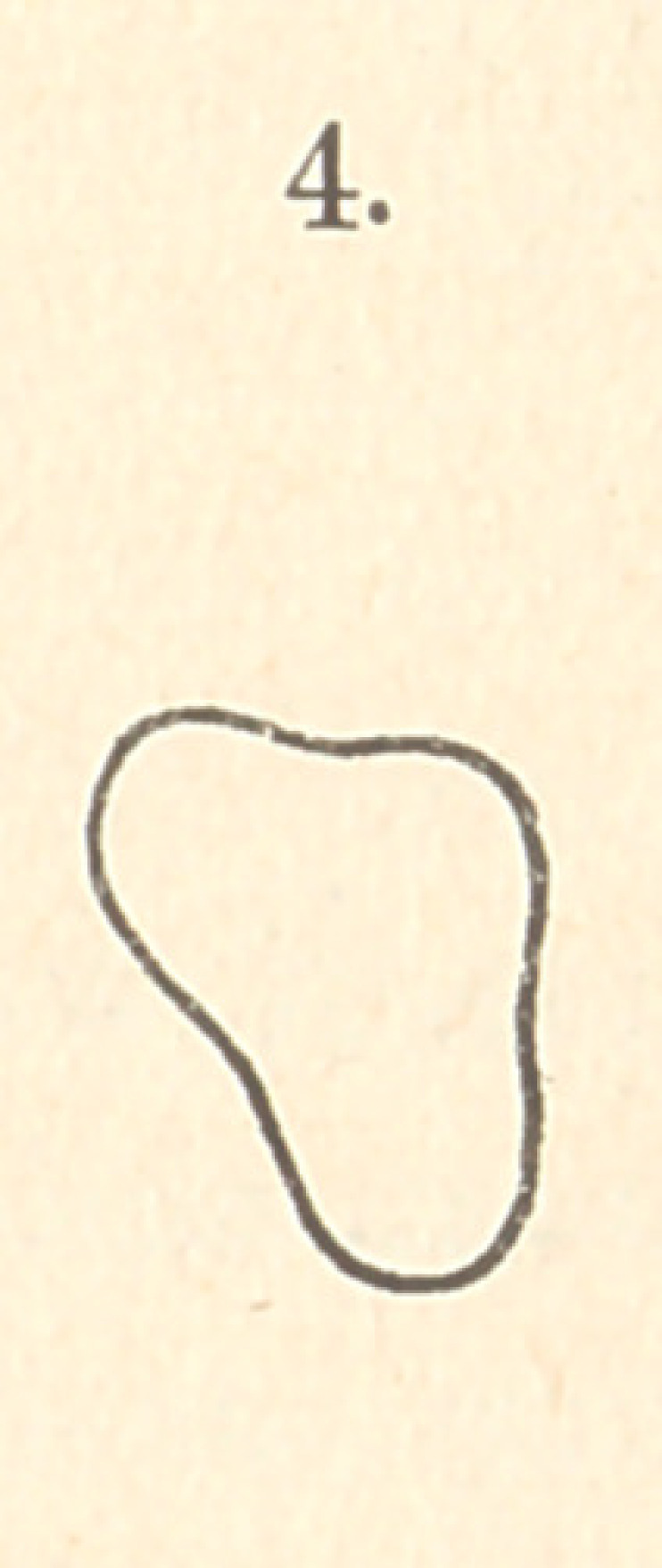


**Fig. 5. f5:**
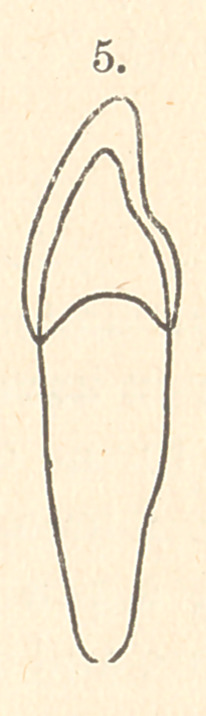


**Fig. 6. f6:**
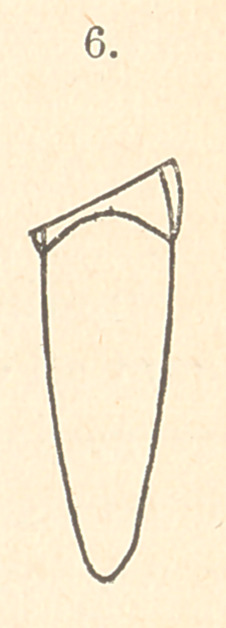


**Fig. 7. f7:**
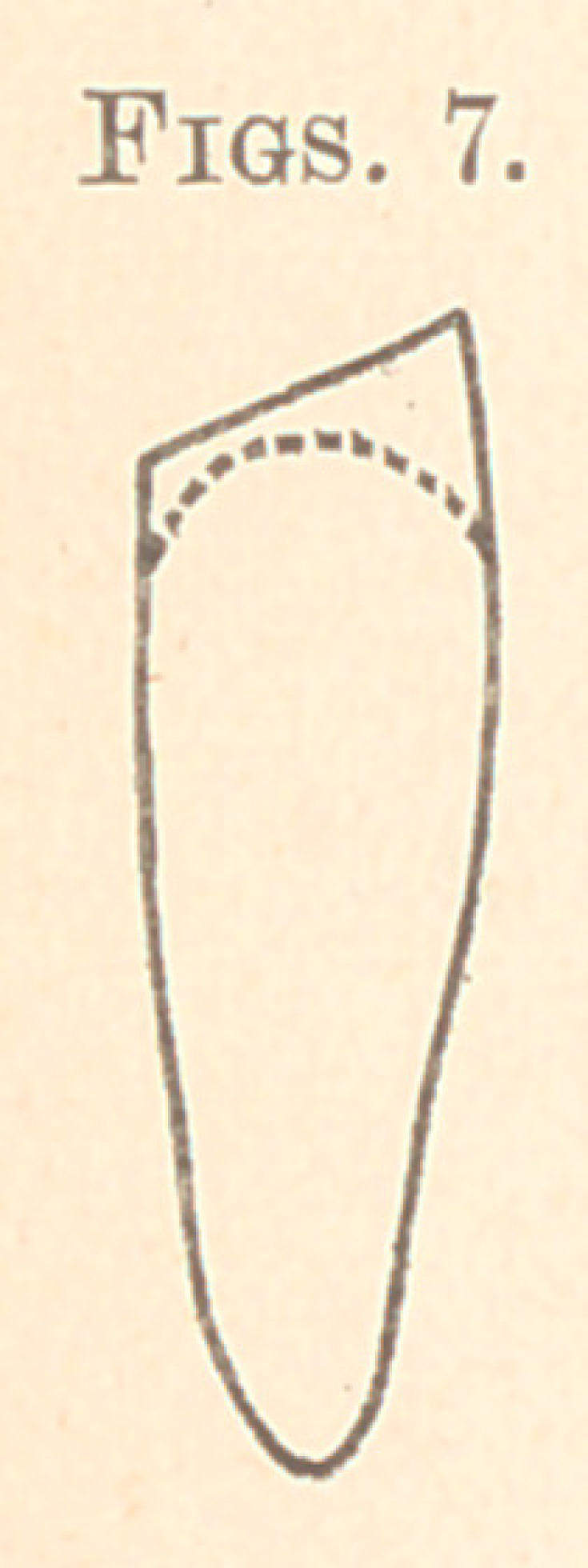


**Fig. 8. f8:**
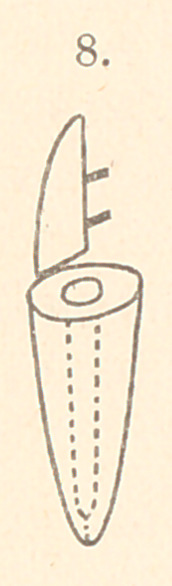


**Fig. 9. f9:**
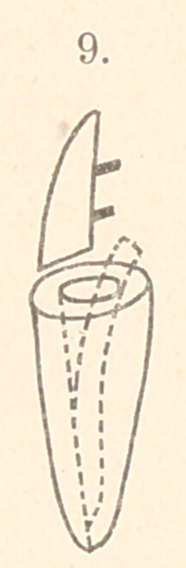


**Fig. 10. f10:**
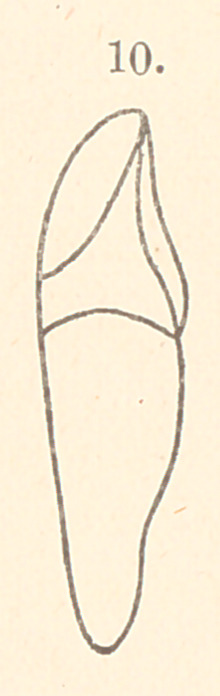


**Fig. 11. f11:**
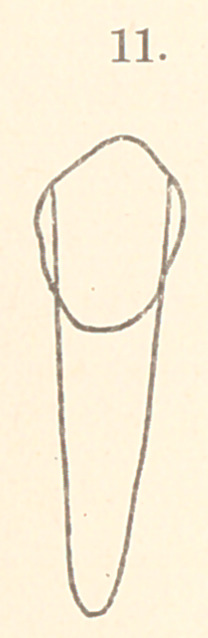


**Fig. 12. f12:**
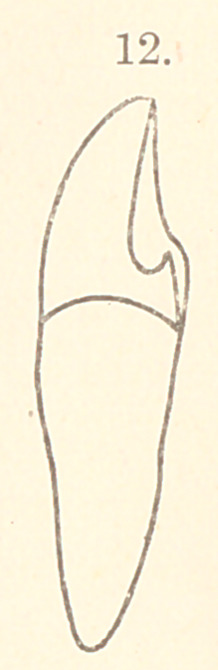


**Fig. 13. f13:**
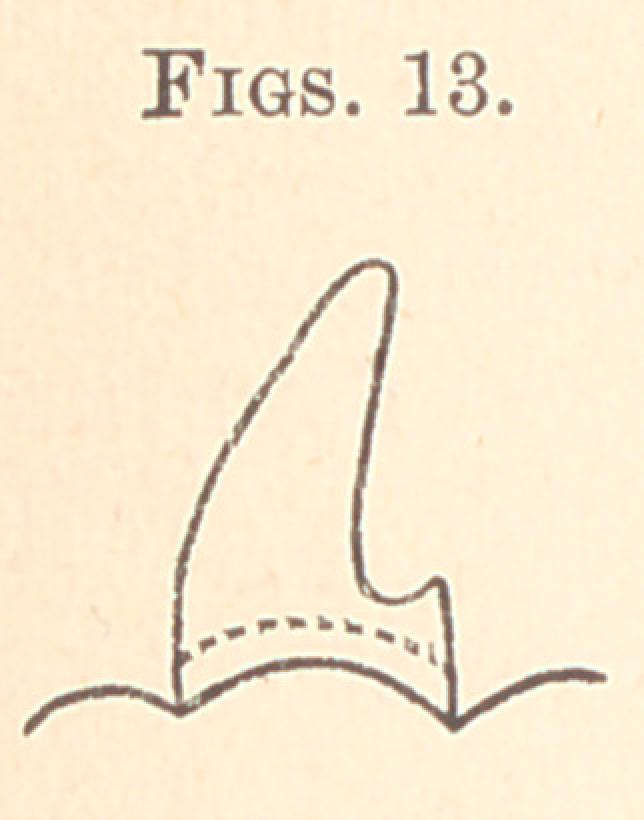


**Fig. 14. f14:**
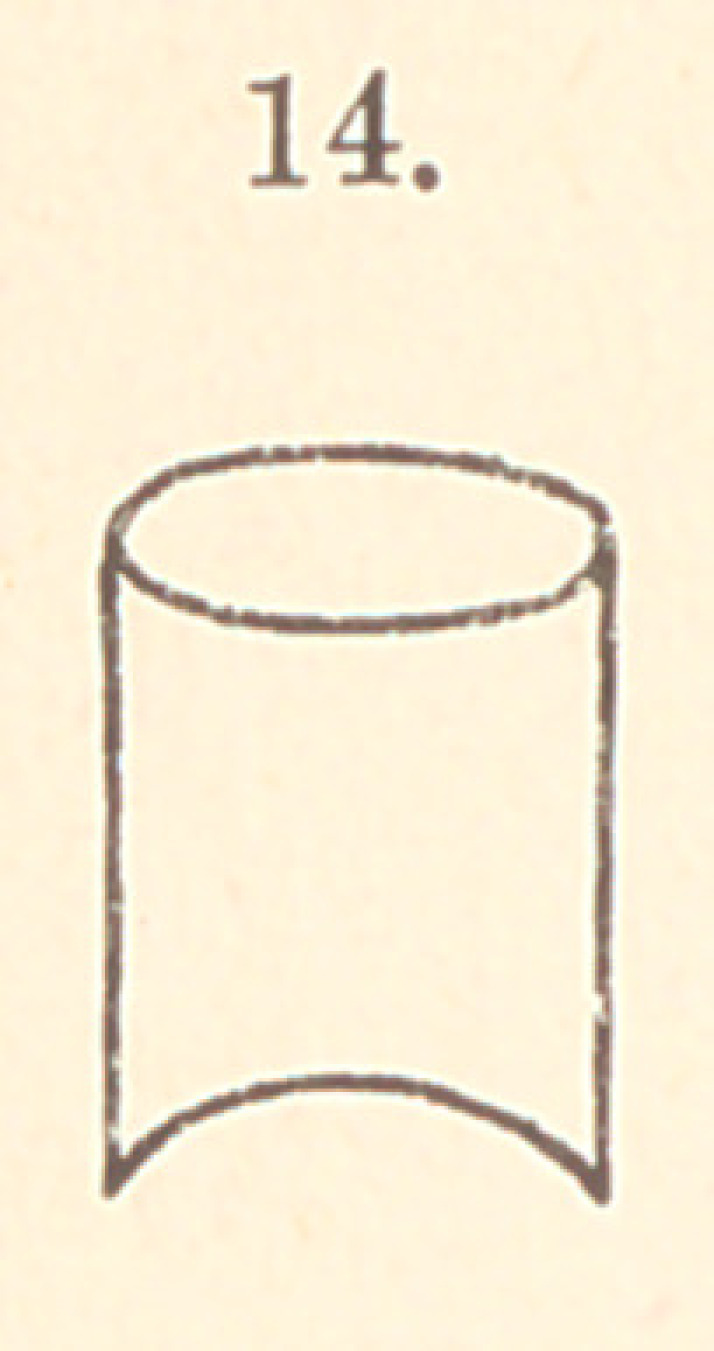


**Fig. 15. f15:**
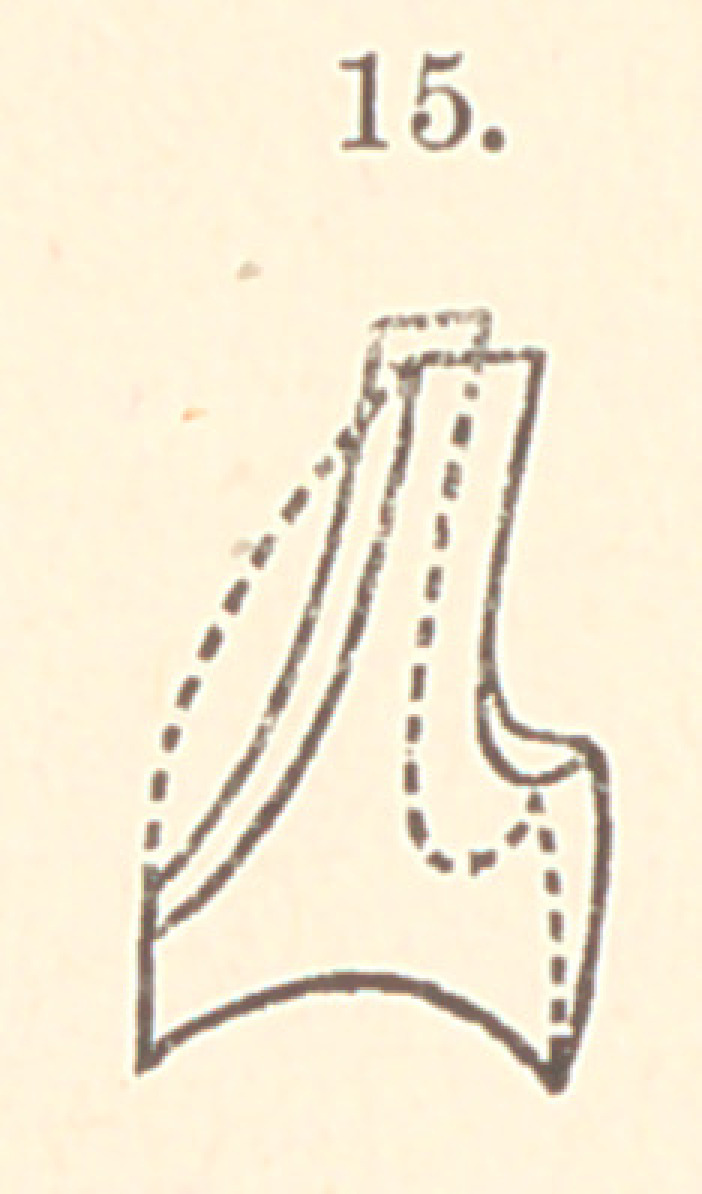


**Fig. 16. f16:**
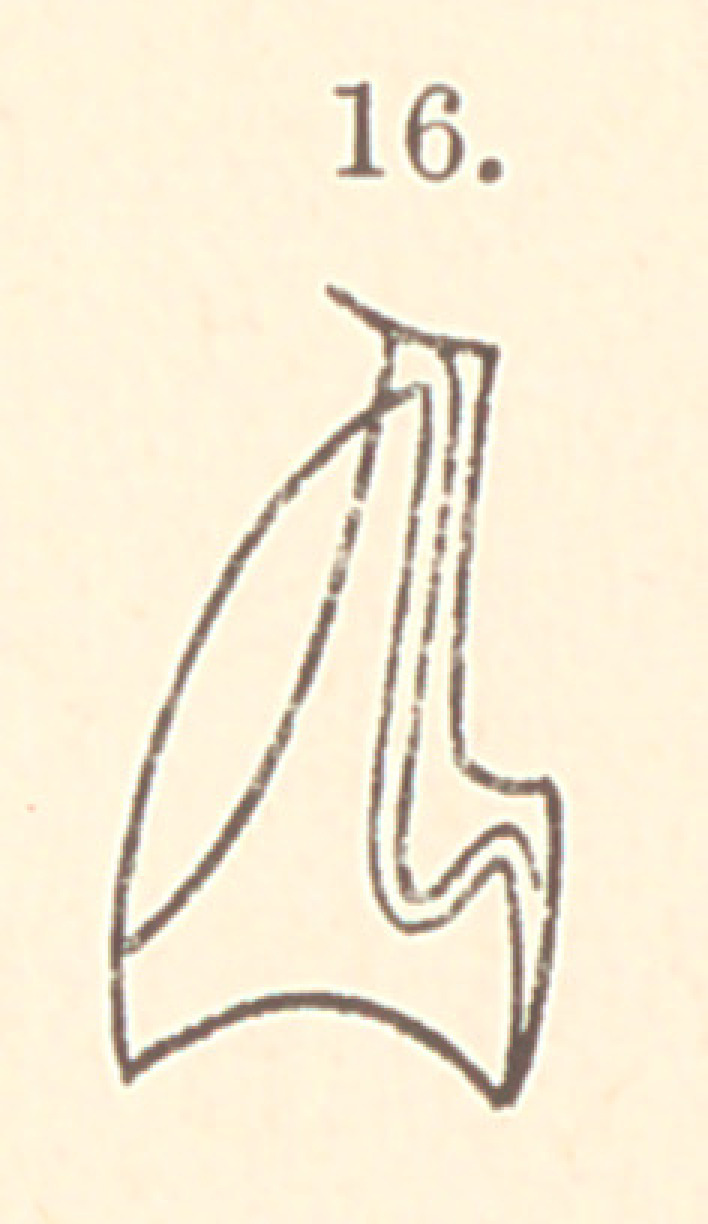


**Fig. 17. f17:**
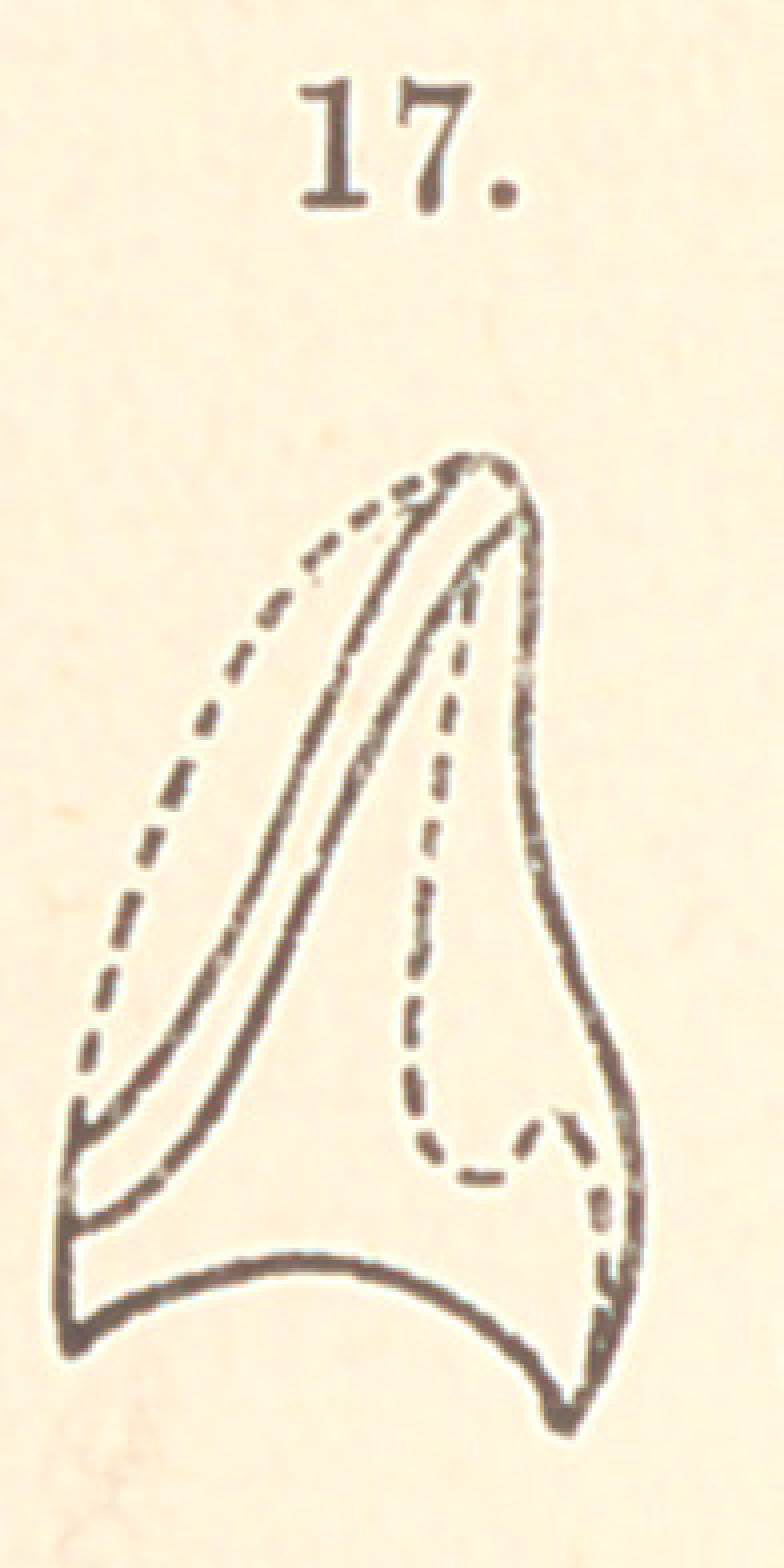


**Fig. 18. f18:**
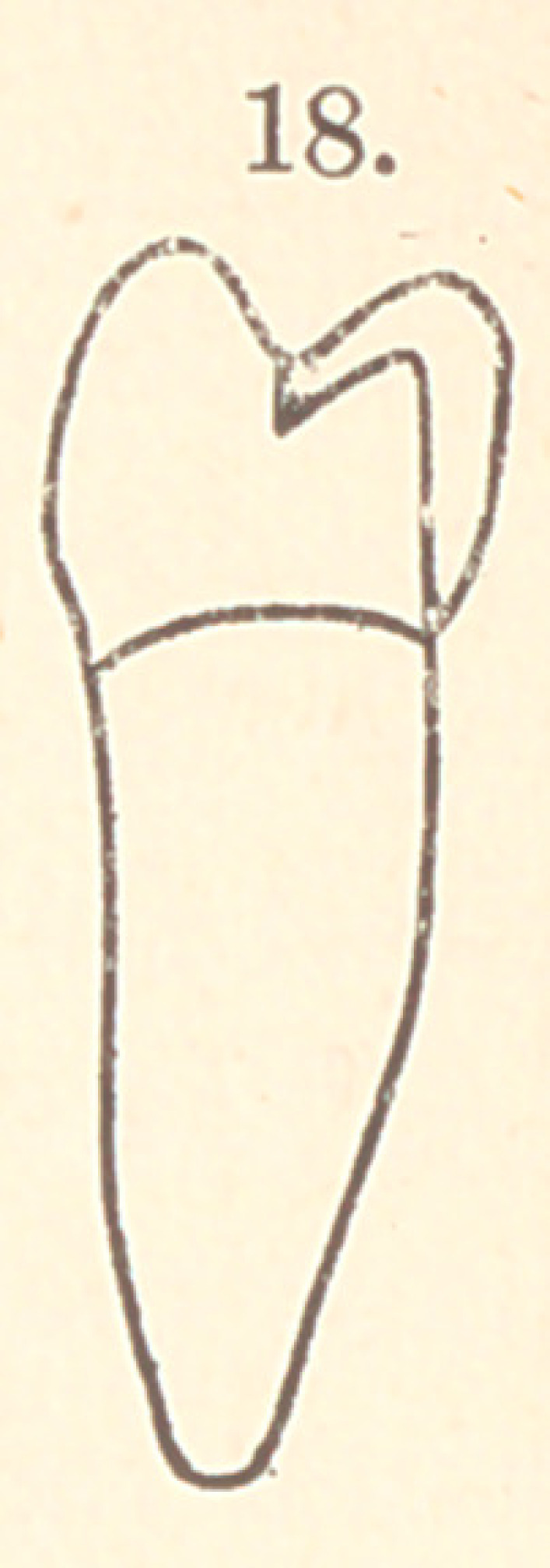


**Fig. 19. f19:**
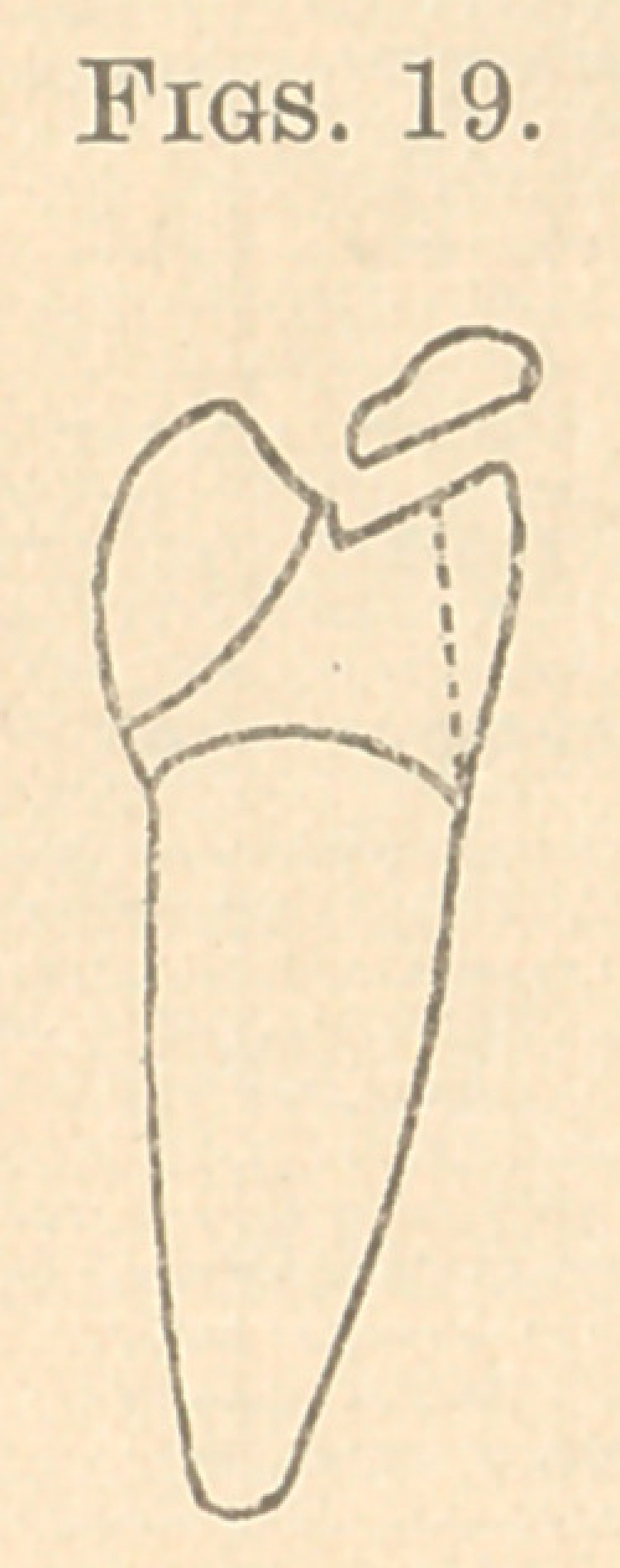


**Fig. 20. f20:**
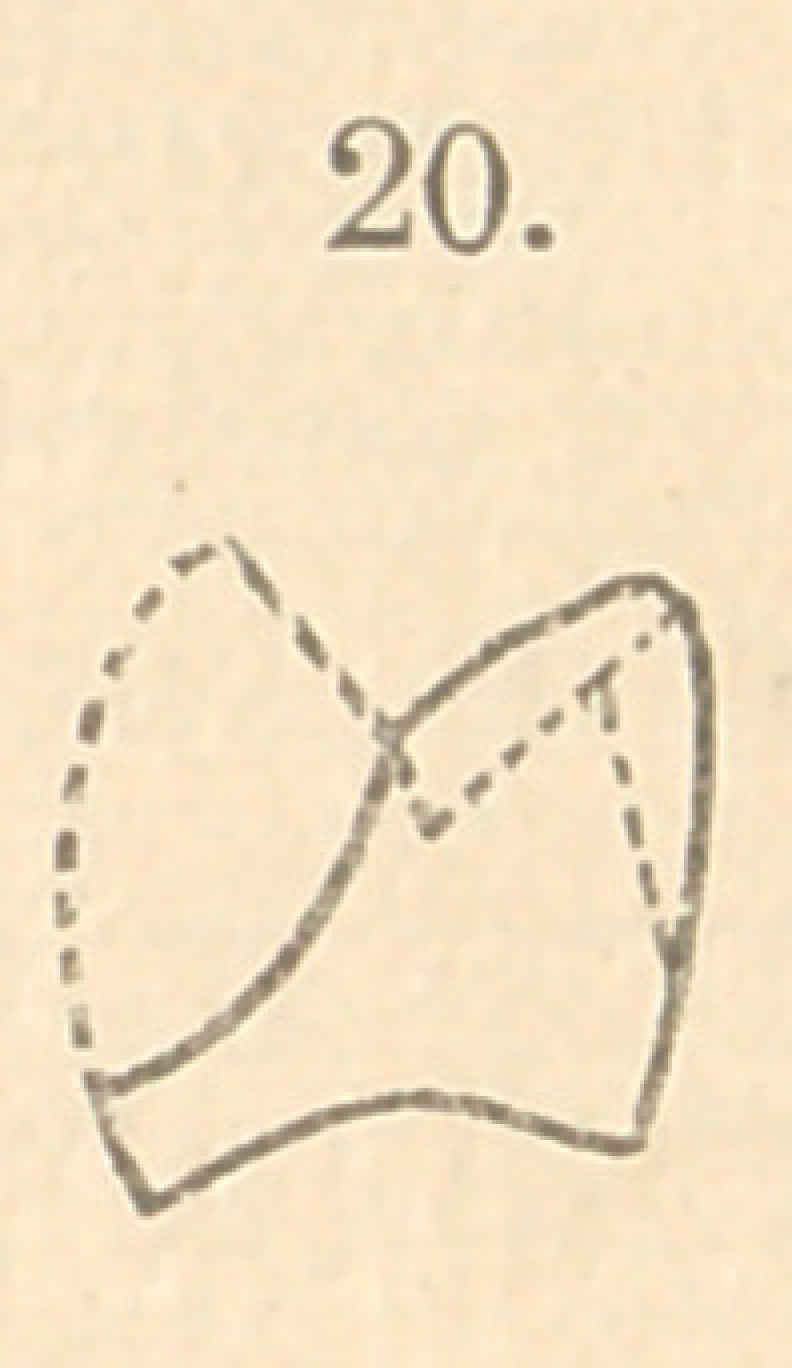


**Fig. 21. f21:**
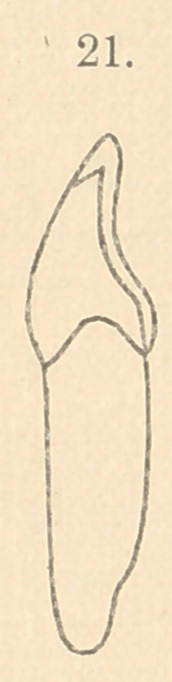


**Fig. 22. f22:**
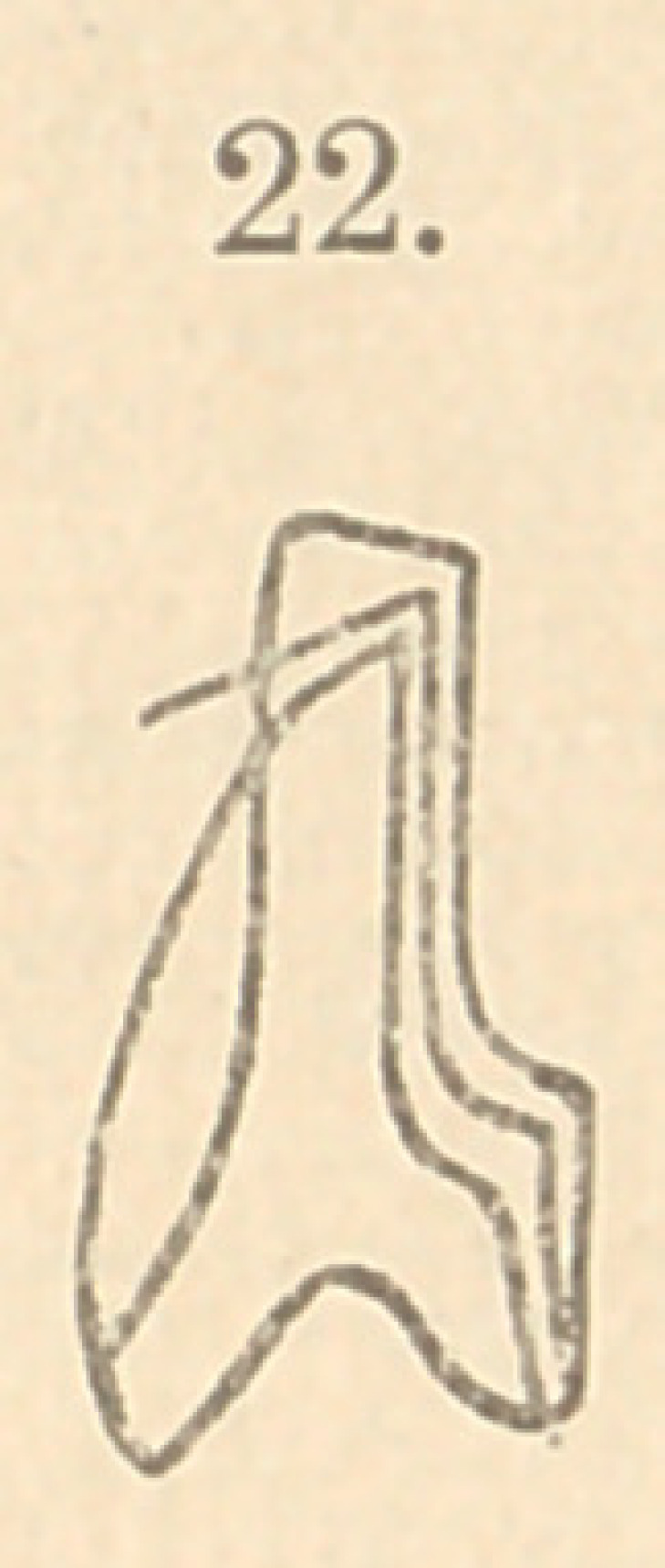


**Fig. 23. f23:**
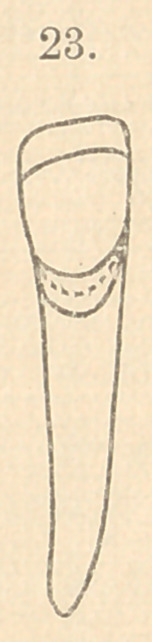


**Fig. 24. f24:**
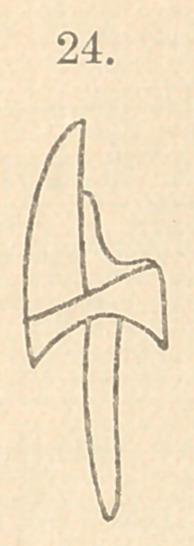


**Fig. 25. f25:**
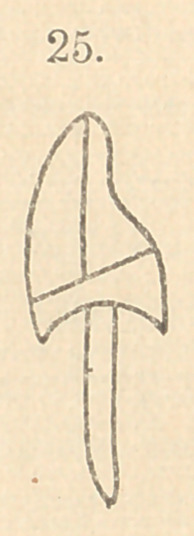


**Fig. 26. f26:**